# Appendiceal adenocarcinoma presenting as left-sided large bowel obstruction, a case report and literature review

**DOI:** 10.1186/1477-7800-4-20

**Published:** 2007-07-27

**Authors:** Moayad M Aljarabah, Neil R Borley, James MD Wheeler

**Affiliations:** 1 Department of Gastrointestinal Surgery, Cheltenham General Hospital, Cheltenham, UK

## Abstract

**Background:**

appendiceal tumours are rare, they may be encountered unexpectedly in any acute or elective abdominal operation, many of these tumours are not appreciated intraoperatively and are diagnosed only during formal histopathological analysis of an appendicectomy specimen. Herein we present a case of appendiceal adenocarcinoma presenting as left-sided large bowel obstruction, we also review the literature of unusual presentations of appendiceal tumours.

**Case Presentation:**

we report a case of left sided large bowel obstruction found to be secondary to an appendiceal adenocarcinoma. The patient presented with abdominal pain, distension and constipation, CT scan showed large bowel obstruction thought to be due to a sigmoid tumour, on laparotomy the appendix was also noted to be abnormal. A low Hartman's was performed with en-bloc total hysterectomy and bilateral salpigo-oophorectomy. A separate ileocaecal resection with end ileostomy was also performed, pathology specimens showed that the primary neoplasm was the appendix with metastasis to the distal sigmoid.

**Conclusion:**

appendiceal tumours are rare, they usually present as acute appendicitis, other presentations are far less common.

## Background

Neoplasms of the vermiform appendix are rare, they account for 0.4 – 1% of all gastrointestinal malignancies [1]. In a review of over 2000 appendicectomy specimens, histological confirmation of appendiceal neoplasm included carcinoid (0.27 %), adenocarcinoma (0.14%), malignant mucocele (0.005 %) and lymphoma (0.005 %) [2]. They are more common in men, with the highest incidence in the fifth decade of life [3].

Most symptomatic appendiceal tumours present as appendicitis, other presentations have also been described, but are far less common, these tumours may also be encountered unexpectedly in any acute or elective abdominal operation and the correct diagnosis is rarely made pre or intraoperatively, most of these tumours are identified only after histopathological examination of an appendicectomy specimen (some 0.7 – 1.7% of appendicectomy specimens contain an appendiceal neoplasm [1].

Herein, we report a case of appendiceal adenocarcinoma presenting as left sided large bowel obstruction, we also present a literature review of the unusual presentations of appendiceal tumours.

## Case presentation

### Case report

An 80 year old female patient was admitted as an emergency with abdominal pain, distension and constipation. Her past medical history included mild hypertension. On admission she was afebrile, with a BP of 146/79, and pulse of 110. On examination of the abdomen she had distension with lower abdominal tenderness. Abdominal XR showed dilated small and large bowel loops. The WCC (White Cell Count) was raised at 12.6, with CRP of 54 & bilirubin of 34. A CT scan of the abdomen showed large bowel obstruction thought to be secondary to a sigmoid carcinoma. A solid mass in the right adnexa was also noted and thought to be either a primary ovarian tumour or metastatic deposit. No distant disease was seen.

After a period of resuscitation, a laparotomy was performed. The large bowel obstruction was found to be secondary to a "sigmoid tumour". The uterus and right ovary were abnormal and were "fixed" to the upper rectum distal to the tumour. The appendix was noted to be abnormal with palpable ileocaecal lymph nodes. A low Hartman's was performed with en-bloc total hysterectomy and bilateral salpigo-oophorectomy. A separate Ileocaecal resection with end ileostomy was also performed. Postoperatively the patient made an uneventful recovery and she was discharged home two weeks later.

Macroscopic examination of the specimen showed an appendix measuring 40 mm in length and up to 15 mm in diameter. Microscopic examination showed invasive high grade adenocarcinoma of appendiceal origin showing diffuse infiltration of the wall of the appendix and the caecum with serosal extension. Metastatic tumour was seen in 2 out of 9 ileocaecal lymph nodes. Distant metastatic carcinoma was also shown to involve the wall of the distal sigmoid colon with stricture formation and infiltration of the adherent uterus, cervix, both ovaries and right fallopian tube.

**Figure 1:**
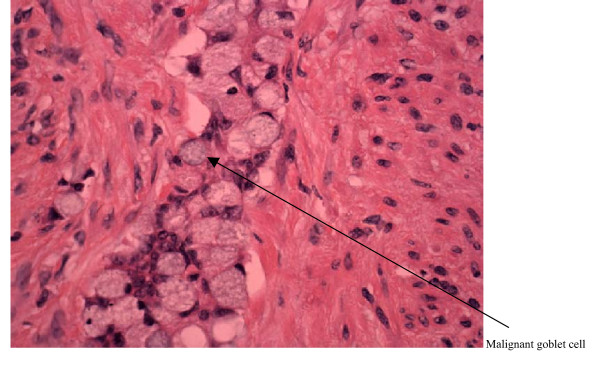
400× goblet cell mucinous adenocarcinoma of the appendix.

Immunohistochemistry showed that in all areas of tumour infiltration identical staining was identified. There was strong CK20 positivity, patchy chromogranin positivity and negative staining for CK7. There was similarity in appearance to a malignant goblet cell carcinoid although the tumour was classified as a high-grade aggressive adenocarcinoma of the appendix with neuroendocrine differentiation (see figure 1).

The patient was started on Chemotherapy, but 2 weeks into her treatment she declined any further Chemotherapy as she felt very unwell with it

Two years postoperatively the patient remains well with a clear surveillance CT scan.

### Literature review

In our review of the literature, some other unusual presentations of appendiceal tumours have also been reported, they included an appendiceal adenocarcinoma presenting as a vesical fistula [4], neck mass and vaginal bleeding [3], spontaneous skin fistula [5], caeco-colic intussusception [6], disseminated ovarian carcinoma [7]. There was also a reported case of adenocarcinoma of the appendix masquerading as a bladder tumour [8], a uterine tumour [9], and a case presenting as an inguinal hernia [3].

## Discussion

Clinically most appendiceal tumours present as acute appendicitis (79.1% in one series reported [10]), another report [11] described the clinical presentations of 74 patients found to have appendiceal tumours, some 49% presented as acute appendicitis, 9.5% were incidental findings, 5.4% presented as pelvic abscesses, 6.4% with gastrointestinal symptoms, and 6.4% with bowel obstruction. Other less common presentations (1.4% each) included carcinoid syndrome, RIF (Right Iliac Fossa) mass, inflammatory bowel disease and strangulated hernia.

Appendiceal neoplasms could be found at any acute or elective abdominal surgery, management plan should then be based on the intraoperative findings. Murphy et al [1] suggested that for tumours found incidentally at operation, if the tumour was confined to the appendix, smaller than 2 cm, without evidence of mesoappendiceal involvement and not involving the base of the appendix, appendicectomy is appropriate. Any neoplasm greater than 2 cm and any involving the base of the appendix or mesoappendix should be considered for immediate right hemicolectomy for an optimal outcome. For patients who are found to have appendiceal neoplasm on histopathological examination, if the lesion is benign or carcinoid less than 2 cm and confined to the appendix, then appendicectomy alone is sufficient. Those who present with a perforated, though macroscopically removed, epithelial tumour should have baseline tumour markers (CEA, CA-125, CA-19-9), CT scan and colonoscopy.

Depending on the stage and the grade of the tumour, the overall 5-year survival reported for adenocarcinoma of the appendix is in the region of 55 percent [2]. Radiotherapy in general has no role in the treatment unless the margins are involved and like colorectal cancer, Chemotherapy is offered to patients with positive lymph nodes.

Appendiceal neoplasms are also noted to be associated with a significant incidence of synchronous and metachronous colorectal neoplasms [1]. Patients with colorectal cancer have 3–5 percent risk of synchronous and 2–3 percent risk of metachronous neoplasia [12], and a recent report has observed similar incidences of synchronous and metachronous tumours of the appendix and the colon. The authors go on to suggest that because of the inability to assess the appendiceal mucosa postoperatively, the routine removal of the appendix in patients undergoing colorectal cancer resection is justified [12].

## Conclusion

Appendiceal tumours are rare, they usually present as acute appendicitis but can be encountered unexpectedly in any acute or elective abdominal operation. An unexpected clinical presentation should be considered by clinicians.

As part of the formal exploratory laparotomy, in addition to the routine thorough examination of the abdominal viscera, looking for deposits and lymphadenopathy, we suggest the inspection and palpation of the appendix and mesoappendix as part of the intraoperative examination of any gastrointestinal malignancy surgery.

## Competing interests

The author(s) declare that they have no competing interests.

## Authors' contributions

MA: Collected data, wrote the provisional manuscript

NB: Helped with the design of the manuscript, revised the contents of the paper

JW: General supervision, given final approval of the version to be submitted

All authors read and approved the final manuscript.
